# Cardiac Myosin Binding Protein-C Plays No Regulatory Role in Skeletal Muscle Structure and Function

**DOI:** 10.1371/journal.pone.0069671

**Published:** 2013-07-31

**Authors:** Brian Lin, Suresh Govindan, Kyounghwan Lee, Piming Zhao, Renzhi Han, K. Elisabeth Runte, Roger Craig, Bradley M. Palmer, Sakthivel Sadayappan

**Affiliations:** 1 Department of Cell and Molecular Physiology, Health Sciences Division, Loyola University Chicago, Maywood, Illinois, United States of America; 2 Department of Cell Biology, University of Massachusetts Medical School, Worcester, Massachusetts, United States of America; 3 Department of Molecular Physiology and Biophysics, University of Vermont, Burlington, Vermont, United States of America; Texas A & M, Division of Cardiology, United States of America

## Abstract

Myosin binding protein-C (MyBP-C) exists in three major isoforms: slow skeletal, fast skeletal, and cardiac. While cardiac MyBP-C (cMyBP-C) expression is restricted to the heart in the adult, it is transiently expressed in neonatal stages of some skeletal muscles. However, it is unclear whether this expression is necessary for the proper development and function of skeletal muscle. Our aim was to determine whether the absence of cMyBP-C alters the structure, function, or MyBP-C isoform expression in adult skeletal muscle using a cMyBP-C null mouse model (cMyBP-C^(t/t)^). Slow MyBP-C was expressed in both slow and fast skeletal muscles, whereas fast MyBP-C was mostly restricted to fast skeletal muscles. Expression of these isoforms was unaffected in skeletal muscle from cMyBP-C^(t/t)^ mice. Slow and fast skeletal muscles in cMyBP-C^(t/t)^ mice showed no histological or ultrastructural changes in comparison to the wild-type control. In addition, slow muscle twitch, tetanus tension, and susceptibility to injury were all similar to the wild-type controls. Interestingly, fMyBP-C expression was significantly increased in the cMyBP-C^(t/t)^ hearts undergoing severe dilated cardiomyopathy, though this does not seem to prevent dysfunction. Additionally, expression of both slow and fast isoforms was increased in myopathic skeletal muscles. Our data demonstrate that i) MyBP-C isoforms are differentially regulated in both cardiac and skeletal muscles, ii) cMyBP-C is dispensable for the development of skeletal muscle with no functional or structural consequences in the adult myocyte, and iii) skeletal isoforms can transcomplement in the heart in the absence of cMyBP-C.

## Introduction

**Figure 1:**
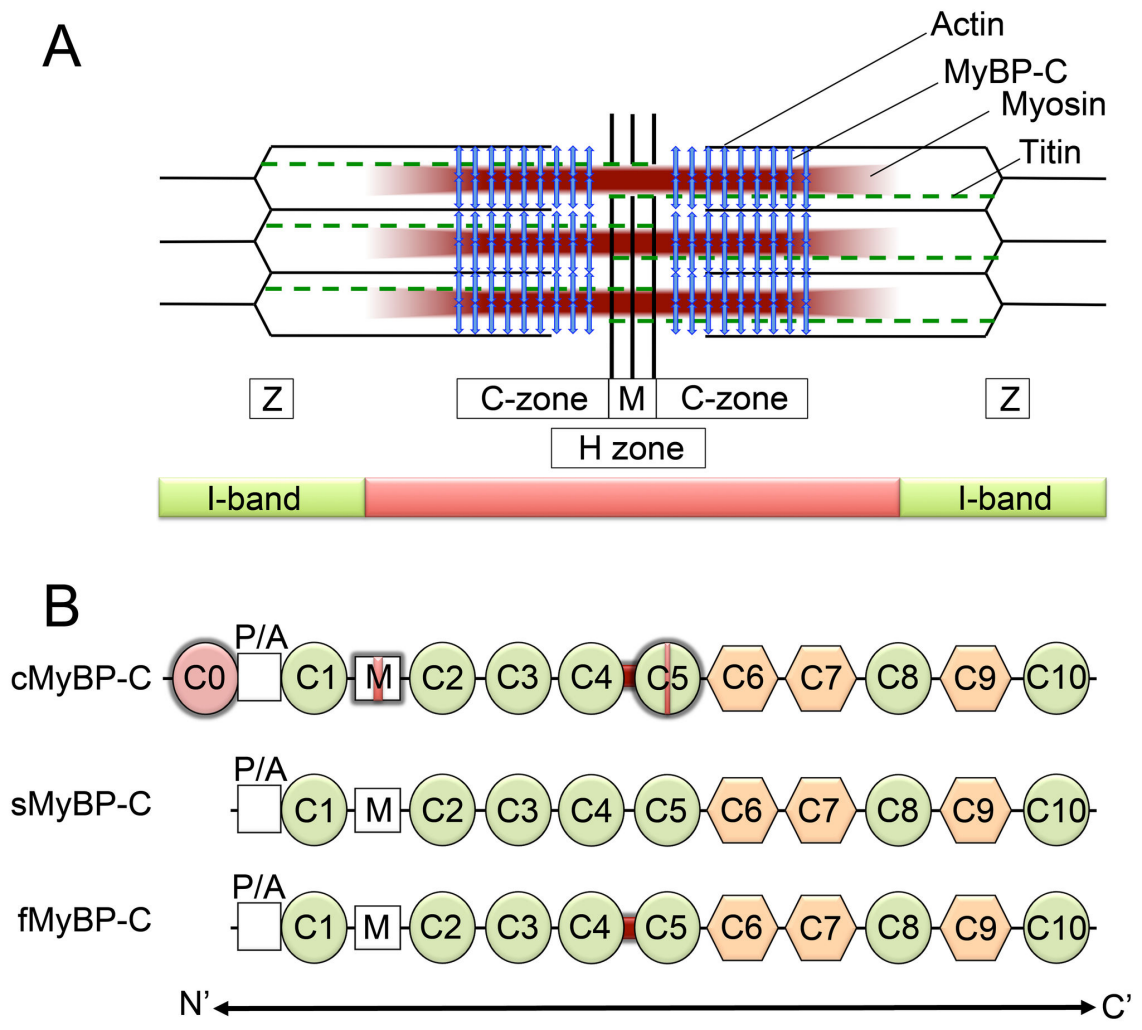
Schematic diagram of striated muscle sarcomere and the three main isoforms of MyBP-C. (**A**) The bulk of MyBP-C is oriented perpendicularly to the long axis of the myosin filaments and is restricted to the C-zone in the central 1/3 of each half A-band (vertical arrows). Titin (dashed lines) is a giant protein that spans the length of the half-sarcomere. MyBP-C is restricted to the C-zone of the A-band and connects both thick and thin filaments [1]. (**B**) The three main isoforms of MyBP-C: a cardiac form (cMyBP-C) and two skeletal isoforms, slow and fast (sMyBP-C and fMyBP-C, respectively). MyBP-C isoforms each have a proline–alanine (P/A)-rich region towards the N-terminus, three fibronectin type III domains (hexagons), and seven immunoglobulin domains (circles). The cardiac isoform has an additional immunoglobulin domain, C0, at the N-terminus, phosphorylation motifs in the M domain (red vertical band), and a twenty-eight residue cardiac-specific insert in the C5 immunoglobulin domain (red vertical band). The cardiac and fast skeletal isoforms share a conserved ‘linker’ between the C4 and C5 domains (dark thick band). Distinctions between the different isoforms are highlighted.

Myosin binding protein-C (MyBP-C) is a modular thick filament protein belonging to the intracellular immunoglobulin (Ig) and fibronectin (Fn) superfamily (Figure 1). It is found in vertebrate cardiac and skeletal muscle and has both regulatory and structural functions [1,2]. It is localized in 7-9 axial stripes, 43 nm apart, in the middle one-third (the “C zone”) of each half A-band [3]. The localization and arrangement of these stripes are highly conserved among different MyBP-C isoforms [4]. MyBP-C can bind myosin at two sites; the N-terminal region binding to S2 [5] and to the regulatory light chain [6], and the C-terminus binding to light meromyosin [7]. MyBP-C can also bind to actin [4] [8], as well as to other thick filament proteins, such as titin [9]. There are 3 isoforms of MyBP-C, each encoded by a distinct gene: fast-skeletal, slow-skeletal, and cardiac [10–13]. The cardiac isoform (cMyBP-C) differs from the skeletal isoforms in that cMyBP-C has an extra Ig domain at the N-terminus (C0), four phosphorylation sites within a MyBP-C specific domain known as the M-domain, and a 28 residue insert in the C5 Ig module [14] (Figure 1). The two skeletal isoforms of MyBP-C expressed in mature skeletal muscles are slow MyBP-C (sMyBP-C) and fast MyBP-C (fMyBP-C), encoded by the *MYBPC1* and *MYBPC2* genes, respectively (Table 1). *MYBPC1* is expressed in both slow and fast skeletal muscles, whereas *MYBPC2* is expressed only in fast skeletal muscle [12]. The cMyBP-C gene (*MYBPC3*) is predominantly expressed in cardiac muscle [15,16]. Mutations in MyBP-C lead to myopathy in both skeletal and cardiac muscles. *MYBPC1* mutations cause distal arthrogryposis type 1 [17], while mutations in *MYBPC3* are associated with the development of hypertrophic cardiomyopathy (HCM) [18,19]. Mouse models lacking cMyBP-C exhibit pronounced cardiomyopathies, including HCM [20] and dilated cardiomyopathy (DCM) [21].

**Table 1:** Summary of differences in gene location, expression, and known associated myopathies among the three major isoforms of MyBP-C.

	**sMyBP-C**	**fMyBP-C**	**cMyBP-C**
Gene symbol	*MYBPC1*	*MYBPC2*	*MYBPC3*
Chromosome location (human)	12	19	11
Chromosome location (mouse)	10	7	2
mRNA size (mouse)	3941 base pairs	3610 base pairs	4224 base pairs
Protein length (mouse)	1127 amino acids	1136 amino acids	1270 amino acids
Expression Profile(adult tissue)			
Heart	Yes	No	Yes
Skeletal Muscle	Yes	Yes	No
Diseases	Distal arthrogryposis type I	No skeletal myopathies currently associated	Hypertrophic cardiomyopathy; dilated cardiomyopathy; skeletal myopathy

Database reference: www.ncbi.nlm.nih.gov

While skeletal and cardiac MyBP-C are expressed primarily in their corresponding tissues, expression of skeletal MyBP-C in cardiac tissue and of cMyBP-C in skeletal tissues can also occur. In addition to its expression in skeletal muscles, sMyBP-C is also expressed in the right atrium and the interatrial septum of the heart [22]. Likewise, during avian embryogenesis, the cMyBP-C gene, *MYBPC3*, is expressed in skeletal muscle in addition to its expression in cardiac muscle [23]. This skeletal expression of cMyBP-C in birds occurs early in embryonic development, followed by later expression of skeletal isoforms [24]. Since it is formed at the same time as myosin and titin, cMyBP-C is hypothesized to be necessary for the formation of skeletal sarcomeres during myofibrillogenesis. These studies suggest that i) cMyBP-C is necessary for skeletal muscle development, and ii) its ablation could therefore alter adult skeletal muscle structure and function. While cMyBP-C expression in developing avian skeletal muscle is well established and has also been observed in amphibians [25], comparable evidence for cMyBP-C in developing mammalian skeletal muscle has been lacking [15,16]. Nevertheless, two intriguing observations suggest that this possibility should not be discounted. Mutations in *MYBPC3*, normally associated with the development of HCM [18,19], have also been demonstrated to cause skeletal myopathy, apparently due to expression of mutated cMyBP-C in the skeletal muscle [26]. Furthermore, in studies of human skeletal myoblast-to-myotube transitions, cMyBP-C transcript was present [27]. However, cMyBP-C protein was not detected by immunohistochemistry, which is not very sensitive for detecting proteins.

These observations have prompted us to further explore the possible significance of cMyBP-C in developing skeletal muscle of mammals, particularly whether the absence of cMyBP-C affects normal skeletal muscle structure and function [16]. We have assessed the expression of MyBP-C isoforms, the structure of the skeletal muscles, and the functional characteristics of slow (soleus) and fast (extensor digitorum longus, EDL) skeletal muscles in the cMyBP-C null mouse model (t/t) ) [21] to test whether i) cMyBP-C is necessary for normal adult skeletal muscle structure, ii) DCM-induced myopathy leads to alteration in MyBPC expression, and iii) transcomplementation of skeletal MyBP-C isoforms compensates for the absence of cMyBP-C in the heart, compared to the wild-type (WT) control mice. Our data show that absence of cMyBP-C does not induce significant changes in isoform expression in skeletal muscle, nor does it produce any significant structural or functional deficit in skeletal muscle, indicating that cMyBP-C is dispensable for normal muscle development. However, there is an increase in fMyBP-C, but not sMyBP-C, in the heart.

## Results

### MyBP-C isoform expression patterns in adult striated muscle

**Figure 2:**
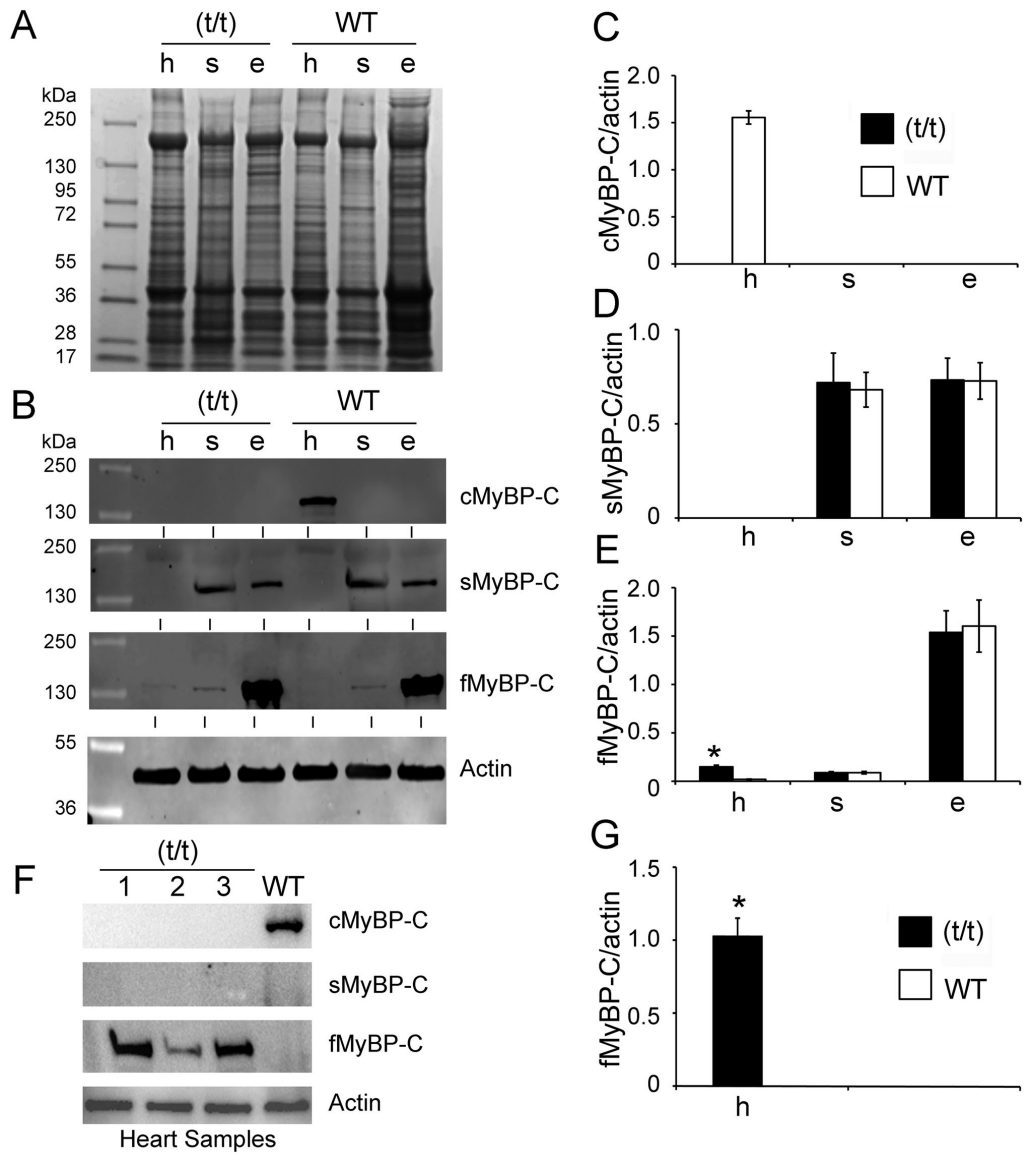
MyBP-C isoforms are differentially expressed in cardiac and skeletal tissue. Ten µg of total proteins were resolved on 4-15% SDS-PAGE and stained with Coomassie Brilliant Blue (**A**). Expression levels of cardiac, slow skeletal and fast skeletal MyBP-C in (t/t) and WT mice were determined in heart (h), soleus (s) and EDL (e) muscles by Western blot analysis with respective antibodies (**B**). Representative Western blot shows that the presence of cardiac isoform of MyBP-C (cMyBP-C) is exclusive to ventricular muscle of WT mice (**C**), but completely absent in the (t/t) mouse hearts. sMyBP-C was significantly expressed both in the soleus muscle and EDL muscle (**D**). Conversely, fMyBP-C was mainly detected in EDL muscle (**E**), but significantly increased in the (t/t) hearts. All values were normalized to the expression of α-sarcomeric actin (MyBP-C/actin ratio) and expressed as relative values. The summarized quantitative data were derived from n=3 with mixed gender mice (*p< 0.01 versus WT). The increased level of fMyBP-C expression in the (t/t) hearts was reconfirmed by Western blot analysis (**F**) and not found in the WT hearts, summarized in panel **G** (n=6, *p< 0.0001 versus WT). α-sarcomeric actin was used as a loading control.

To determine the differential expression of MyBP-C isoforms in adult muscle, extracts of cardiac and skeletal tissue from (t/t) and WT mice were probed by Western blotting for the slow, fast, and cardiac isoforms of MyBP-C, using antibodies known to be specific for these isoforms. cMyBP-C was not detected in either slow or fast skeletal muscle tissues (Figure 2A–C), confirming that cMyBP-C expression is restricted to the adult heart. Strong expression of sMyBP-C was determined to be present in both the soleus and EDL. Prominent expression of fMyBP-C was detected in the EDL of both (t/t) and WT mice, but detectable quantities were also found in soleus muscles of (t/t) and WT mice. In contrast with studies indicating that sMyBP-C is natively expressed in the right atria and interatrial septum of other species [22], we did not detect sMyBP-C in whole heart homogenates of our mice. Strikingly, fMyBP-C expression level was significantly increased in the (t/t) hearts, compared to the WT ventricles (Figure 2B, EF & G). In order to increase the anti-fMyBP-C antibody sensitivity, samples were reanalyzed without skeletal muscle controls for fMyBP-C (Figure 2F). As expected, fMyBP-C expression was markedly increased in the (t/t) hearts, compared to the WT hearts, which displayed no fMyBP-C expression (Figure 2G). These data suggest that in adults, cMyBP-C is exclusive to the heart and not expressed in WT and DCM-induced HF skeletal muscles.

**Figure 3:**
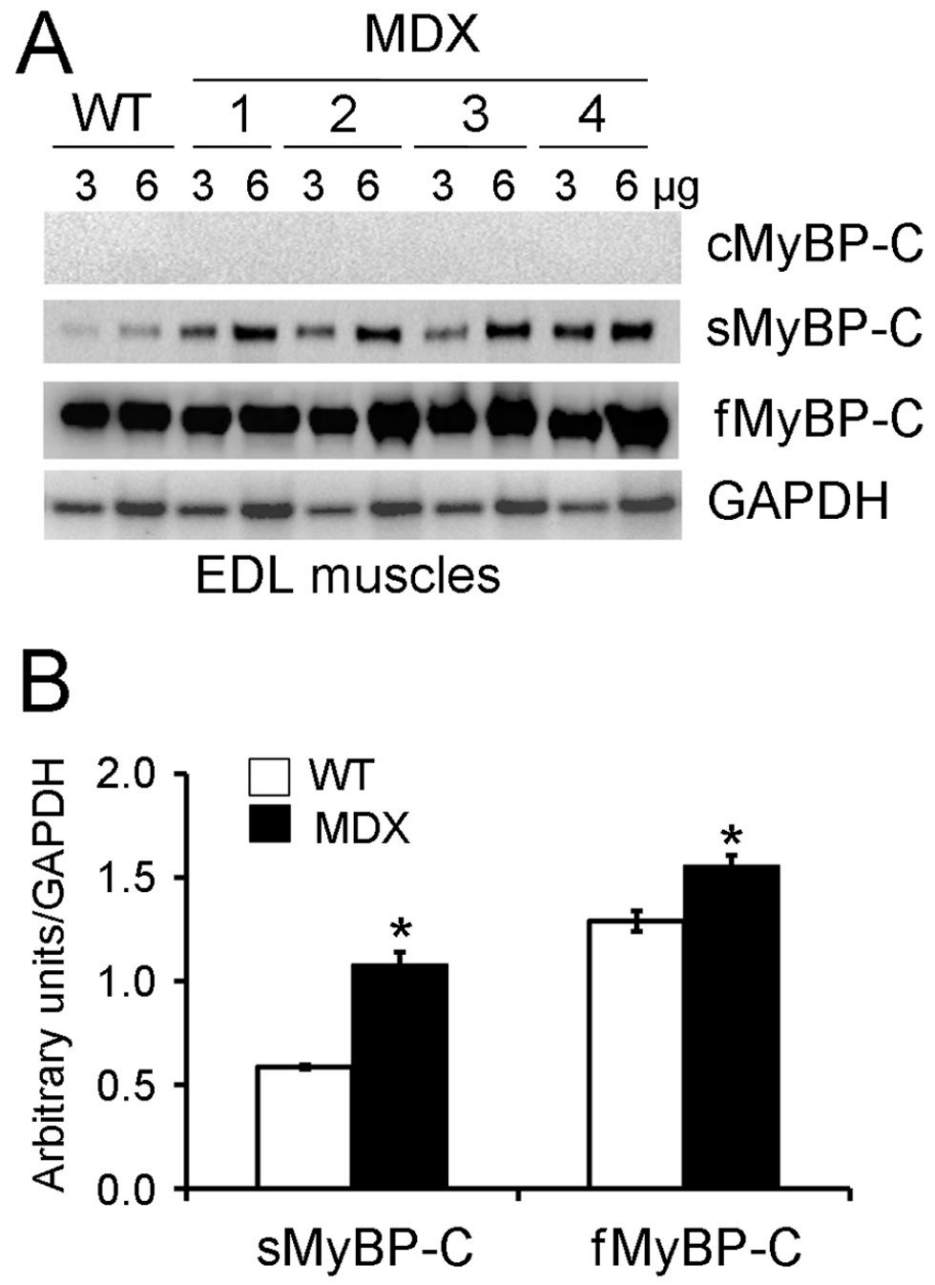
cMyBP-C was not expressed in myopathic skeletal muscles. To determine the expression level of MyBP-C isoforms in myopathic fast skeletal muscles, three and six µg of total EDL muscle proteins from both WT and *mdx* mice were loaded into SDS-PAGE followed by immunoblotting using respective MyBP-C antibodies (**A**). The quantitative data were summarized in panel B (n=4, *p<0.01 versus WT). GAPDH was used as a loading control.

Dystrophic skeletal muscles of *mdx* mice (a model of human Duchenne muscular dystrophy) display significant degeneration-regeneration cycles that are reminiscent of developing myocytes. To assess whether cMyBP-C might be expressed in skeletal muscles as part of these cycles, dystrophic skeletal muscles of *mdx* mice were analyzed. EDL fast muscle samples were collected from 6-month old WT and *mdx* mice and analyzed for the expression of sMyBP-C, fMyBP-C, and cMyBP-C by Western blot analysis. Results showed that cMyBP-C was not expressed in the skeletal myopathy muscles (Figure 3). In contrast, sMyBP-C and fMyBP-C were both significantly increased in the *mdx* muscles. Taken together, these data suggest that in the adult 1) cMyBP-C is exclusively expressed in cardiac, but not skeletal, tissue of either WT or *mdx* mice, 2) fMyBP-C expression is increased in the absence of cMyBP-C in (t/t) mouse hearts, and 3) increased expression of sMyBP-C or fMyBP-C could be a marker of skeletal myopathy. Furthermore, our data confirm that sMyBP-C is expressed in slow and fast skeletal muscles, while fMyBP-C is predominantly expressed only in fast skeletal muscles.

### cMyBP-C is dispensable for development of normal adult muscle structure

**Figure 4:**
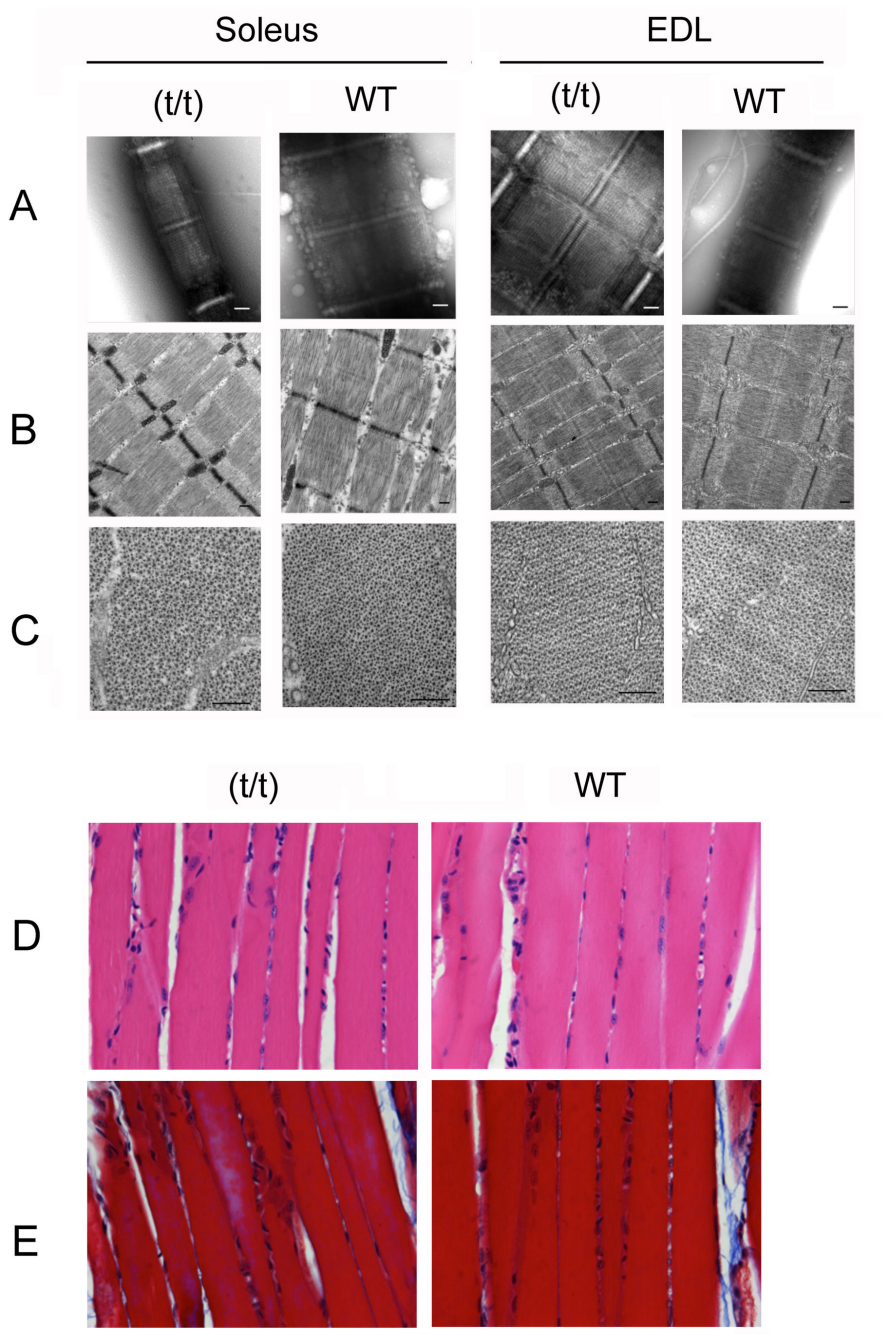
Ultrastructural and histopathological analyses show similar sarcomeric structure in (t/t) and WT soleus and EDL skeletal muscles. Phenotype of mouse soleus (slow) and EDL (fast) muscle myofibrils observed by negative staining (**A**) and thin sectioning (**B** and **C**). The structure and organization of sarcomeres in both longitudinal section (B) and transverse section (C) are not disrupted in either soleus or EDL in (t/t) mice. A-band length, M-line width and Z-disc width confirm electron microscopy images, showing no significant change between (**t/t**) and WT mice (Table 2). I-band length variations are likely due to different lengths of the muscle during fixation. Scale bars = 200 nm. Representative hematoxylin and eosin (**D**) and Masson’s trichrome (**E**) staining images show no detectable changes in myofibrils or fibrosis in the (t/t) soleus muscles, compared to the WT control.

To determine whether ablation of cMyBP-C in the (t/t) mice alters sarcomere structure in skeletal muscles, electron microscopy analyses were performed using slow (soleus) and fast (EDL) skeletal muscles from (t/t) and WT mice. Muscles were studied by negative staining of isolated, fixed myofibrils and by longitudinal and transverse sectioning of fixed and embedded specimens (Figure 4 A–C). Results showed no apparent difference between (t/t) and WT mice in any of the skeletal muscles tested. Soleus and EDL muscles showed typical A-bands, I-bands, M-lines, and Z-lines in both sections and myofibrils, with no significant difference in lengths between WT and (t/t) (Table 2). In the negatively stained specimens, transverse stripes were often very prominent in the A-bands of both (t/t) and WT muscles. The stripes most likely represent cardiac troponin, based on their ~38 nm periodicity measured in Fourier transforms and their appearance in the I-band in some cases. Though MyBP-C stripes can sometimes be visualized in myofibrils and sections, they were not apparent in these specimens, even in the WT muscle. Therefore, we cannot draw any conclusions about possible effects of cMyBP-C ablation on the MyBP-C stripes in (t/t) skeletal muscles. Transverse views of both (t/t) and WT mouse showed the typical hexagonal lattice structure of thick and thin filaments in the A-band (Figure 4 C). Filament diameter and degree of ordering were similar between (t/t) and WT muscles. Other cellular features, such as membranes and mitochondria, were also similar between (t/t) and WT skeletal muscles. Neither trichrome nor H&E staining revealed any differences in gross histology or fibrosis in the skeletal muscles of WT and (t/t) mice (Figure 4 D-E).

**Table 2:** Summarized quantitative values of the sarcomere structure.

		Soleus		EDL	
	(µm)	t/t	WT	t/t	WT
Myofibril	A-band	1.65 ± .02	1.64 ± .01	1.62 ± .01	1.66 ± .04
	M-line	0.13 ± .001	0.12 ± .01	0.12 ± 0.01	0.12 ± .01
	Z-line	0.09 ± 0.01	0.10 ± 0.01	0.11 ± .01	0.09 ± 0.15
Section	A-band	1.44 ± .03	1.48 ± .03	1.44 ± 0.03	1.49 ± .02
	M-line	0.11 ± .01	0.12 ± .01	0.13 ± .01	0.12 ± .01
	Z-line	0.09 ± .01	0.09 ± .01	0.05 ± .01	0.05 ± .01

Average length of A-band and width of M-line and Z-line in (t/t and WT muscles determined by negative staining (Myofibril) and thin sectioning (Section). Measurements of the electron microscopy images were averaged from approximately 10 well-oriented sarcomeres in each case. No significant differences were observed between the (t/t and WT mice in either the soleus or EDL muscles. Shorter A-band lengths in the sections compared with negatively stained myofibrils are consistent with shrinkage effects that are commonly seen during specimen preparation for thin section electron microscopy.

### cMyBP-C ablation does not affect skeletal muscle function

**Figure 5:**
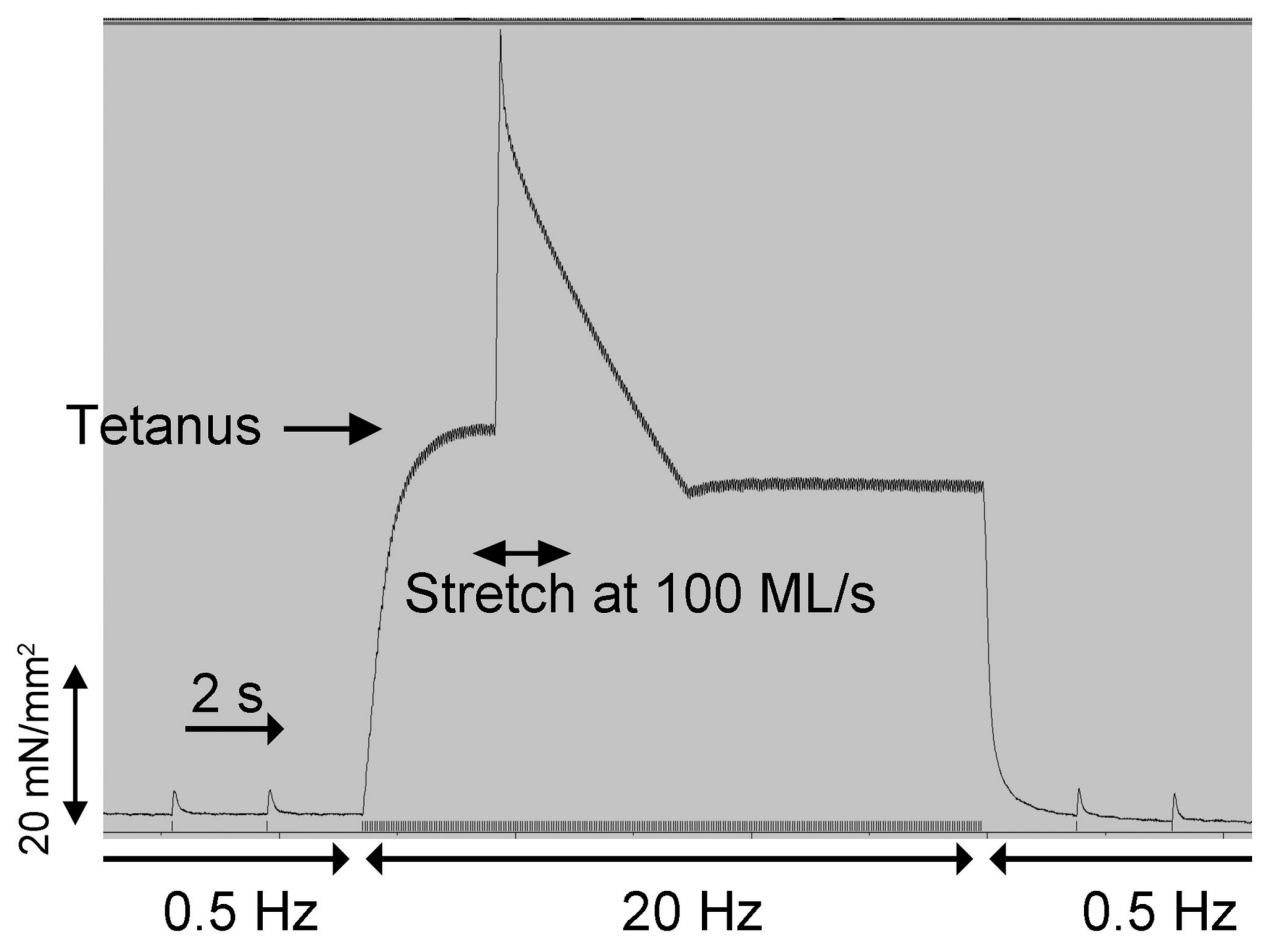
Analysis of twitch and tetanic tension shows no differences between (t/t) and WT mice. Skeletal muscles were attached to a micrometer and force transducer to measure twitch and tetanus tension with mechanical stretches of varying velocities (20, 40, and 60, 100 ML/s) for 100 ms duration (see Methods). Injury from stretch was determined from fractional loss of twitch force prior to tetanus versus post-tetanus stimulation. Stiffness of the muscle was determined from change in force under tetanus. An example tracing from (t/t) soleus muscle is shown here. Measurements from functional analyses of (t/t) and WT are summarized in Table **3**.

To determine whether absence of cMyBP-C during skeletal muscle development and the presence of DCM-induced HF would affect skeletal muscle function in the (t/t) mice, soleus muscles were evaluated for twitch tension, tetanic tension, and injury susceptibility (Figure 5). Fractional loss of twitch force following eccentric contraction was used to estimate injury (Table 3). Results show no difference between (t/t) and WT mice for twitch tension measured prior to tetanus. Twitch tension after eccentric contraction was reduced ~10% in both groups, indicating no increased susceptibility to injury in the (t/t) mice. Tetanus tension was highly variable and not significantly different in either groups. Muscle stiffness (percent of change during stretch) was modestly increased in (t/t) mice in comparison with that of WT mice (p > 0.05, n = 8), indicating a slight, but not statistically significant, increase in stiffness in (t/t) mice. These data suggest that cMyBP-C ablation and presence of DCM-induced HF has no effect on skeletal muscle function at 6 months of age in mice.

**Table 3:** Summarized values of soleus muscle kinetics *in vitro*.

	Twitch Tension (mN/mm^2^)	Recovery after stretch (%)	Tetanus (mN/mm^2^)	Change during stretch (%)
(t/t)	8.2 ± 1.2	88.2 ± 9.4	113.2 ± 13.7	87.8 ± 11.3
WT	6.1 ± 1.8	92.5 ± 6.1	87.2 ± 22.6	63.4 ± 7.5#

Skeletal muscle function was determined in soleus muscles of (t/t and WT mice. Twitch tension was measured after 3V, 7ms electrical stimulation of 0.5Hz, and tetanus tension was measured after 3V, 100ms at 20Hz with mechanical stretches of different velocities applied for 100ms. Twitch tensions were measured post-tetanus, and characteristics of injury from stretch and viscoelastic stiffness were taken from comparisons of fractional loss of twitch force and change in force with the stretches, respectively. The modest change in tetanus during stretch reflects a higher stiffness in the (t/t mice on the order of ~40% compared to WT (n 8 muscles from four mice in each group (#p<0.10)).

### MyBP-C isoforms have distinct promoter regions

Since each MyBP-C isoform is encoded by its own unique gene, we hypothesized that each MyBP-C could be uniquely regulated by tissue-specific transcription factors, which would account for differential tissue-specific expression. We examined putative binding sites of myocyte enhancer factor 2 (MEF2) and the GATA family of transcription factors, both of which are important in normal cardiac and skeletal muscle development, as well as the development of HF [28,29]. To determine whether differential expression of MyBP-C in the heart and skeletal muscles is mediated by unique transcription factors, we used promoter analysis to identify all the transcription factors in mice. Our computational promoter analysis was done using the 4kb upstream regions for putative binding sites of MEF2 and GATA family members, because these are the major transcription factors that regulate cardiac and skeletal muscle specificity. Results show that the binding sites for GATA elements are significantly enriched in *MYBPC3* (70 binding sites), compared to the *MYBPC1* (16 binding sites) and *MYBPC2* (7 binding sites) genes. Binding sites for MEF2 are located in both *MYBPC1* (6 binding sites) and *MYBPC3* (2 binding sites) genes, but absent in the *MYBPC2* gene. The *MYBPC3* promoter overlaps with the last two exons and last intron region of SFPI1, a neighboring gene about 2500 base pairs upstream from *MYBPC3* on mouse chromosome 2. The promoter for *MYBPC2* overlaps with the last exon of SPIB on chromosome 7. Putative binding sites for TTF are found in *MYBPC2* and *MYBPC3* promoters, but they are absent in the *MYBPC1* promoter. *MYBPC2* has two sites within 100 base pairs, while *MYBPC3* has only one site in the 4000 base pairs upstream region we analyzed. A few binding sites for MEF2 were present in both *MYBPC1* and *MYBPC3*, but completely absent in *MYBPC2*. These data support our analysis that isoforms of MyBP-C are differentially expressed in both cardiac and skeletal muscles.

## Discussion

In this study, we determined whether cMyBP-C is necessary for the proper development and function of skeletal muscle, as appeared possible from studies of avian species and from certain observations on mammalian skeletal muscle [23,24,27,30]. We investigated the differential expression of MyBP-C isoforms in cardiac and skeletal muscles in mice lacking cMyBP-C and in dystrophic mice. Our studies confirm that cMyBP-C is completely ablated in the (t/t) mice and is never expressed in adult skeletal muscles of WT or myopathic (*mdx*) mice, whether the tissue is slow type (soleus) and fast type (EDL). Furthermore, we show that cMyBP-C is completely dispensable for normal structure and function of skeletal muscle and that fMyBP-C, while expressed in the heart in the (t/t) mouse, does not prevent dysfunction. These data show that transient expression of cMyBP-C in developing skeletal muscle is not required for normal adult skeletal muscle function and that other isoforms are not upregulated in skeletal muscle to compensate for the lack of cMyBP-C.

Our study is in good agreement with previous reports indicating that cMyBP-C does not transcomplement in adult skeletal muscle [15,16,27]. In addition, (t/t) mice skeletal muscle did not exhibit any structural nor functional abnormalities, indicating that cMyBPC is dispensable for normal muscle development. Studies of avian muscle have shown that cMyBP-C expressed during embryonic skeletal muscle development plays a critical role in myofibrillogenesis [23,24,30]. While mammalian studies argue against a comparable role for cMyBP-C in mammals, observations of skeletal myopathy in an infant with a truncating *MYBPC3* mutation [26], and the finding of cMyBP-C transcripts in proliferating human skeletal mononucleated myoblasts and myotubes [27] have suggested that this possibility should be kept open. Our study determined that global cMyBP-C ablation in (t/t) mice showed no structural or functional deficits in skeletal muscles, and we confirmed that cMyBP-C is neither expressed in normal adult skeletal muscle nor induced as a result of skeletal myopathy in *mdx* mice. Thus, expression of cMyBP-C in mammals is restricted to cardiac muscle and does not transcomplement in skeletal muscles. cMyBP-C appears to be dispensable for adult skeletal muscle structure and function; early embryonic expression of cMyBP-C in myoblasts may be ectopic [27], without any specific role in adult skeletal muscle.

Our results confirm that sMyBP-C is significantly expressed in both slow and fast skeletal muscles, whereas fMyBP-C is predominantly expressed only in fast skeletal muscle [27,31]. sMyBP-C is the first isoform to be expressed in both mice and humans during skeletal muscle development, followed by fMyBP-C in fast skeletal muscles [15,31]. Moreover, sMyBP-C and fMyBP-C are co-expressed and co-exist in the same muscle type and sarcomere [16,22,31,32]. Interestingly, sMyBP-C, which is expressed in the right atrium and interatrial septum of adult vertebrate heart [22], has four variants that are differentially spliced and regulated in skeletal muscles [33]. However, it is unknown whether skeletal MyBP-C isoforms are upregulated in the heart either due to absence of the cardiac isoform or presence of HF. In the case of troponin, troponin I and T have slow, fast, and cardiac isoforms [34,35]. The cardiac and skeletal isoforms are expressed in both skeletal and cardiac muscles during development, and the skeletal isoforms are upregulated in the heart during HF [34,36], indicating that transcomplementation occurs. Skeletal myosin heavy chains (isoforms I, IIa, and IIx) [37] and α-skeletal actin [38] are also expressed in the heart during the development of HF. Urboniene et al. (2005) showed that expression of slow skeletal troponin I in the heart improves cardiac function [39]. These studies suggest that expression of skeletal sarcomeric protein isoforms in the heart during HF can be beneficial. Therefore, we determined the expression of skeletal MyBP-C isoforms in hearts that did not express cMyBP-C. We found that fMyBP-C was significantly increased in the (t/t) mouse hearts compared to WT. In contrast, sMyBP-C was expressed in both (t/t) and WT hearts in atria, but not ventricles. We found that the expression levels of both sMyBP-C and fMyBP-C skeletal isoforms were unaltered in (t/t) mouse skeletal muscles compared to the WT mouse control, suggesting that ablation of cMyBP-C does not alter skeletal isoform expression. However, both skeletal isoforms were significantly increased in the *mdx* skeletal muscles, as described previously [16]. Interestingly, promoter analysis supports our hypothesis that MyBP-C isoforms are differentially regulated in both cardiac and skeletal muscles. Specifically, GATA binding sites, but not MEF2 sites, are enriched in the *MYBPC3* gene; in contrast, low abundance of MEF2 and GATA sites are present in *MYBPC1*. These studies further show that the enriched level of GATA binding sites in *MYBPC3* indicates its specific expression in cardiac and not skeletal muscles. However, it should be noted that these binding sites are computationally predicted and therefore may or may not be functional [40,41]. Taken together, our analysis demonstrates that skeletal isoforms are differentially regulated in the heart and skeletal muscles.

Skeletal myopathies are diseases that affect skeletal muscles, either by inherited genetic defects or concomitant with endocrine, inflammatory, or metabolic disorders [42]. Inherited myopathies, including periodic paralysis, muscular dystrophies, and congenital myopathies, are caused by genetic defects that lead to the absence of essential functional proteins or the presence of modified proteins that cause skeletal muscular defects. Furthermore, patients with HF often develop reduced skeletal muscle function, manifesting itself as exercise intolerance [43,44]; and are often at risk for developing non-cardiac deficits, including skeletal muscle myopathies [45,46]. While some studies show that insults to the heart do not appear to alter skeletal muscle structure and function [47], other evidence suggests that cardiac myopathies do adversely affect skeletal muscle function in humans [48]. To further understand the relationship between heart failure and skeletal muscle function, we investigated whether DCM-induced HF in (t/t) mice altered the ultrastructure or function of the skeletal muscles, compared to the WT control. Despite disruption of both structure and function in cardiac tissue in the (t/t) mice [21], electron microscopy and histology both revealed that the absence of cMyBP-C in (t/t) mice had no effect on the structure or organization of myofilaments in either fast or slow skeletal muscles. Furthermore, no functional differences were observed in the (t/t) skeletal muscles compared to WT mice. The lack of functional phenotype in skeletal muscle due to lack of cMyBP-C may be in part explained by the varying etiology, severity, and individual physiological responses to a dysfunctional heart, i.e. some patients may develop secondary dysfunction in the skeletal muscles while others only present symptoms specific to the heart. Tissues from (t/t) mice did not exhibit any statistically significant difference from WT mice in twitch tension, tetanus, and percent of recovery after stretch. However, percent of change during stretch was slightly elevated in (t/t) mice, indicating that (t/t) mice may have slightly increased skeletal muscle stiffness, in accordance with previous data from heterozygous mice (+/t) that demonstrated a similar trend (unpublished data). In the case of the heterozygous mice, fibers had to be stretched to a longer total length to reach a 2.2 µm sarcomere length compared to the WT fibers. In the absence of structural changes in electron micrographs or histological sections, our study cannot determine whether the trend in increased stiffness (p<0.10) is physiological or an artifact of the experimental setup in the (t/t) mice. Studies indicate that the duration of stress on peripheral tissues imposed by HF is an important factor leading to dysfunction [45]. Chronic HF has been shown to decrease skeletal muscle blood flow, but this is not immediately detrimental due to compensatory mechanisms. Eventually these mechanisms fail, especially with the introduction of additional stressors, such as exercise or advanced age [49]. The addition of stressors was not tested in our experiments. While it is possible that the skeletal muscle of (t/t) mice was not sufficiently challenged, we did allow enough time for DCM to manifest in the (t/t) mice and ample time after the development of DCM to observe any downstream effects. Future studies will determine whether potential structural and functional changes in the skeletal muscle of (t/t) mice may be induced by exposing skeletal muscle to additional stressors, such as exercise or β-adrenergic stimulation.

In conclusion, our study demonstrates that in mice, cMyBP-C is exclusively expressed in the heart and not skeletal muscles. In contrast, sMyBP-C is expressed in the right atrium and the outflow track of myocardium, and fMyBP-C is expressed in the ventricles in the absence of cMyBP-C in the heart, such as during DCM-induced HF. We determined that ablation of cMyBP-C does not affect the structure and function of either slow or fast skeletal muscles in 6-month-old mice, underscoring its role specifically in cardiac muscles. Furthermore, lack of cMyBP-C does not contribute to skeletal muscle dysfunction and myopathy and that cMyBP-C is dispensable in the development of skeletal muscle. Systematic studies using mouse models are underway to determine whether isoform expression during embryogenesis is affected by the absence of cMyBP-C and whether fMyBP-C or sMyBP-C can compensate for the cMyBP-C deficit and heart failure. Finally, it will be important to determine whether fMyBP-C expression in the heart has any functional role in altering cardiac contractility.

## Materials and Methods

### cMyBP-C^(t/t)^ Mouse Model

Six-month-old knock-in mouse model (cMyBP-C^(t/t)^ or (t/t)), in which a homozygous mutation in *MYBPC3* causes a C’-modified cMyBP-C that does not incorporate into sarcomeres, resulting in a null cMyBP-C sarcomere [21] and non-transgenic FvB/N wild-type (WT) mice were used. The (t/t) mouse on a FvB/N background has been previously characterized to study the functional deficits of cMyBP-C protein in the heart [50–53]. Evidence of cardiac myopathy development can be detected within six weeks of birth in (t/t) mice, and functional deficits can be measured at ten weeks of age. C57BL/6J and C57BL/10ScSn-*Dmd* ^*mdx*^/J (*mdx*) mice were obtained from The Jackson Laboratory and were housed at Loyola University Chicago [54]. The present animal experiments were specifically approved by the protocols of the Institutional Animal Care and Use Committees at Loyola University Chicago (LU #202172, IACUC 2013008) and the University of Vermont College of Medicine (UVM IACUC #10-019) and followed the guidelines of the *Guide for the Use and Care of Laboratory Animals* published by the National Institutes of Health.

### Western Blot Analysis

Detection of slow MyBP-C (sMyBP-C), fast MyBP-C (fMyBP-C), and cMyBP-C proteins in cardiac and skeletal muscles was done with 4-20% SDS-PAGE (mini precast protein gels, Bio-Rad, Hercules, CA) and Western blotting as previously described [55]. Tissue extracts from left ventricle, soleus, and EDL were homogenized in urea buffer (50mM Tris-HCL pH 7.5, 4M urea, 1M thiourea, 0.4% CHAPS, 20mM permine and 20 mM DTT). Homogenates were quantified using Quick Start^TM^ Bradford protein assay (Bio-Rad, Hercules, CA) and ~10µg of each sample were visualized with Coomassie Blue stain (0.25g R-250 Coomassie Brilliant Blue, 125mL isopropanol, 50mL acetic acid and 375mL water). For Western blots, protein bands were transferred to nitrocellulose membranes, detected with Ponceau S staining (0.1% (w/v) in 5% acetic acid (Sigma-Aldrich, St. Louis, MO), blocked with Western Blocking Reagent (Roche, Indianapolis, IN) in T-TBS, and probed with primary polyclonal anti-cMyBP-C^2–14^ antibody [55], monoclonal anti-sMyBP-C antibody (Abcam, Cambridge, MA) and monoclonal anti-fMyBP-C antibody (Sigma-Aldrich, St. Louis, MO), and then the respective secondary HRP-conjugated antibody (1:10000). All immunoblotted membranes were developed with ECL Prime Western blotting detection reagent (GE Healthcare, Pittsburg, PA) and imaged on ChemiDoc XRS (Bio-Rad, Hercules, CA).

### Histochemistry

For histopathological examinations, the muscles were removed from deeply anesthetized mice, drained of blood, and fixed in 10% formalin. The tissue samples were dehydrated through a graded series of alcohols and laid open before being embedded in paraffin. Step-serial sections were taken from 3 samples per group (5 µm). Sections were then stained with hematoxylin-eosin or Masson’s trichrome [55]. The presence of necrosis, myocyte disarray fibrosis and calcification was determined by an expert who was blinded to genotype as previously described [55].

### Electron Microscopy

Muscles were initially fixed by vascular perfusion with glutaraldehyde/paraformaldehyde. Muscles were then dissected, and fixation resumed. For negative staining, myofibrils were isolated from fixed muscles by homogenizing in rigor solution (100 mM NaCl, 3 mM MgCl_2_, 2 mM EGTA, 1 mM NaN_3_, and 5 mM PIPES, pH 7.0), followed by staining with 0.5% ammonium molybdate on a Formvar/carbon-coated grid. For sectioning, fixed muscles were post-fixed with 1% OsO_4_ in 0.1M sodium cacodylate, dehydrated in graded alcohol and then embedded in Epon. Sections (65 nm thick) were cut with a diamond knife and stained with uranyl acetate and lead citrate. A-band length, M-line width, and Z-line width were measured and averaged using ImageJ (National Institutes of Health [NIH]; http://rsbweb.nih.gov/ij/) as described previously [56].

### Functional Analysis

After euthanasia, soleus muscles were removed from mice and placed into Krebs-bicarbonate buffer bubbled with 95% O_2_-5% CO_2_. Platinum omega-shaped clamps were tied to both ends of the muscle, which was then placed between two hooks attached to a length controller and a force transducer. The muscle bath consisted of Krebs-bicarbonate buffer with 2.5 mM Ca^2+^ maintained at pH 7.4 and room temperature. Electrical stimulations of 3 V, 7 ms duration and 0.5 Hz were delivered through the hooks. Muscles were also subjected to tetanus at 20 Hz during which mechanical stretches of varying velocities (20, 40, 60 and 100 ML/s) were applied for 100 ms [57]. The maximal change in force recorded during the stretch was used as an index of the viscoelastic stiffness of the muscle under tetanus. Twitch forces were recorded prior to and after the tetanus activation; fractional loss of twitch force was used to estimate injury by stretch.

### Promoter Analysis

*MYBPC1*, *MYBPC2*, and *MYBPC3* encode for slow skeletal, fast skeletal, and cardiac isoforms of MyBP-C, respectively. The promoter sequences (upstream 4 kb and downstream 1 kb from the transcription start site) of mouse *MYBPC1*, *MYBPC2*, and *MYBPC3* from the UCSC Genome Browser (http://genome.ucsc.edu) were scanned for significantly enriched transcription factor binding sites using the Genomatix Software Suite (http://www.genomatix.de). The p-values were computed using genome-wide promoters as the background set, with p < 0.05 as the cutoff for significance.

### Statistical Analysis

Data were expressed as mean of standard error with data from minimum of three independent experiments (SigmaPlot 9.0). Statistical analyses were performed between the groups using the paired or unpaired Student’s *t*-test. A value of *p*<0.05 was considered statistically significant.
